# Global Prevalence of Nurse Turnover Rates: A Meta-Analysis of 21 Studies from 14 Countries

**DOI:** 10.1155/2024/5063998

**Published:** 2024-06-12

**Authors:** Hui Ren, Pan Li, Yingchun Xue, Wenhao Xin, Xin Yin, Hongyan Li

**Affiliations:** The First Hospital of Jilin University, Changchun, Jilin, China

## Abstract

**Background:**

Nurses represent the largest occupational group within the health care system, comprising half of the global health workforce. Health care settings are facing severe shortages in countries worldwide, with nurse turnover being identified as the primary reason for this shortage. However, estimates of nurse turnover rates vary widely in the relevant literature.

**Objective:**

This meta-analysis aimed to investigate the global nurse turnover rate since 2000 and provide evidence-based assistance to health policy makers and hospital managers.

**Methods:**

A systematic search of the PubMed, Web of Science, Embase, CINAHL, and Cochrane Library databases was conducted for relevant articles from January 1, 2000, to February 1, 2023. This study included cross-sectional, cohort, and longitudinal studies. In the meta-analysis, further risk of bias, heterogeneity, and subgroup analyses were conducted. Stata 17.0 was used for all of the statistical analyses.

**Results:**

In total, 48,157 records were scrutinized in this study, and 21 investigations encompassing 213,314 nurses across 14 countries were eventually included. The global nurse turnover rate ranged between 8% and 36.6%, and the combined nurse turnover rate was 16% (95% confidence interval: 14%–17%). Subgroup analysis demonstrated that the turnover rate was 19% (95% CI: 14%–23%) in Asia and 15% (95% CI: 13%–17%) in North America.

**Conclusions:**

This meta-analysis analysed the literature published from January 2020 to February 2023 and demonstrated that the global nurse turnover rate was 16%. It is suggested that all medical and health institutions actively adopt relevant systems that can reduce the turnover of nurses and promote a more harmonious, healthy, and safe occupational environment for nurses to strengthen the sustainable development capacity of the nurse workforce.

## 1. Introduction

Currently, the global ageing population and the increasing burden of chronic diseases (2020) are putting considerable pressure on the health care system [1]. As the largest occupational group in the health care system, nurses play a key role in the provision of health services [2]. Currently, there are an estimated 200,000 nurses and 20,000 midwives worldwide, thus representing approximately half of the global human resources in the health sector [3]. Nurses account for more than half of Chinese health professionals. They are a major force in enhancing medical reform and improving services to benefit people. With the increasing demand for medical and health services, the global demand for nurses is also increasing rapidly (2022) [4]. Several studies have shown that the world is facing a severe shortage of nurses [4, 5]. From a concerning aspect, WHO has estimated that the global number of nurses will decrease by approximately 7.6 million by 2030 [6]. Some studies have shown that the high turnover rate of nurses is one of the main factors leading to the global shortage of nurses [7, 8].

An increase in nurse turnover will have numerous adverse effects on the health care system. For example, a reduction in the number of experienced professional nurses will increase the lack of human resources and the workload of in-service nurses, thus affecting not only the construction and development of hospital nursing talent teams but also the quality of nursing services and medical safety [9, 10]. In addition, Warshawsky et al. reported that increased nurse turnover leads to an increase in the incidence of falls and stress injuries [11], and increased nurse turnover may even increase the occurrence of adverse outcomes such as patient death [12]. Moreover, the shortage of human resources and the increased turnover of nurses are closely related to the high costs of recruiting and training new nurses [13]. As a result, nurse turnover is being closely evaluated by health policy makers, hospital managers, and health care institutions worldwide [14, 15]. Studies indicate that the consistent and accurate measurement of turnover is a crucial step in solving the problems of the organizational work environment and the management of nursing staff [16]. Therefore, it is imperative to gain a comprehensive understanding of the current state and variations in nurse turnover rates, which would be beneficial in implementing more effective strategies to reduce workforce attrition.

At present, many published cross-sectional studies have mostly included nurse turnover tendency as being the main outcome variable and explored the relationship between one or more groups of factors and nurse turnover tendency [17–20]. However, a greater turnover tendency may not lead to actual turnover, and an exploration of the situations or factors influencing nurses who have actually left their positions may be of more practical value and guiding significance. To the best of our knowledge, there are few internationally published articles on nurse turnover rates. Some recent studies have measured the turnover rate of nurses in emergency departments, ICUs, and obstetrics and gynecology departments. Several studies have also examined the turnover of nurses in specific countries or regions (such as the USA and South Korea). A comparative review of nurse turnover costs reported nurse turnover rates of 44.3%, 26.8%, 19.9%, and 15.1% in four countries, including New Zealand, the USA, Canada, and Australia, respectively [21]. Chen et al. reported that the turnover rate of nurses in Taiwan was 8.9%, which is low [22].

In conclusion, given that the turnover rate varies greatly among countries, it is necessary to assess the global combined nurse turnover rate with scientific methods and comprehensive retrieval strategies. The purpose of this study was to provide relevant information and evidence for health policy makers and hospital managers by conducting a comprehensive search and scientific analysis of the current literature.

## 2. Materials and Methods

This study was completed according to the Preferred Reporting Items for Systematic Reviews and Meta-Analysis (PRISMA) statement. The protocol was registered on PROSPERO (registration number: CRD42023389556).

### 2.1. Search Strategy

The PubMed, Web of Science, Embase, CINAHL, and Cochrane Library databases were searched for related articles from January 1, 2000, to February 1, 2023. To ensure that the relevant literature was collected as comprehensively as possible, a large set of search terms was used. These search terms were developed by using free terms and subject terms and combined with the Boolean operator OR/AND. The search terms included nurse^*∗*^, attrition, leave^*∗*^, turnover, and quit. The utilized search strategy for each database is provided in Appendix A. The exact combination of search terms was (attrition OR leave^*∗*^ OR turnover OR quit) AND (nurse^*∗*^). Furthermore, studies were selected by manually searching the references to determine the comprehensiveness of the search.

### 2.2. Inclusion and Exclusion Criteria

According to the PRISMA statement, the following inclusion criteria, which are shown by using the PICOS framework, were used: participants (P), clinical nurses working in the hospital (nurse practitioners (LPNs)); intervention (I), not applicable; comparison (C), not applicable; outcomes (O), overall turnover rates, or sufficient raw data for calculation; and study design (S), cross-sectional studies, cohort studies, and population-based longitudinal studies.

The exclusion criteria were as follows: (1) meeting, reviews, case reports, letters, or editorials; (2) studies for which the full text was not available; (3) studies with incomplete data; (4) articles published in languages other than English; and (5) studies with a sample size less than 100.

### 2.3. Data Extraction

The study selection process was performed by two investigators (LP and YC). After removing duplicate studies, the titles, abstracts, and full texts of the studies were independently screened by using the inclusion and exclusion criteria. Any disagreement between the two assessors was resolved by discussion with a third evaluator (RH).

The two evaluators independently extracted the following data by using standardized data sheets: author, year of publication, study time, country/region, study type, sampling method, survey method, sample size, turnover rate, data source, and participant characteristics (such as mean age and female ratio). A third evaluator (RH) verified the extracted data.

### 2.4. Risk of Bias Assessment

Two researchers used the bias risk assessment tool that was developed and designed by Hoy and colleagues to determine the internal and external validity of prevalence studies [23], aiming to evaluate the quality of the included studies. This evaluation tool contains two-item subscales (external validity and internal validity), totalling 10 items. Each item is scored as 1 (“yes,” “high quality”) or 0 (“no,” “low quality”). The total score for a study is pooled from all of the item scores. The total possible scores are 9, 6–8, and 0–5, thus representing high, medium, and low quality, respectively. Low-quality studies had a high risk of bias; therefore, they were excluded from this study. The risk of bias assessment was conducted by LP and counterchecked by YC, with discrepancies resolved by RH.

### 2.5. Data Synthesis

The turnover rate (*p*) was calculated via the following equation:

(1) $$\begin{array}{c} \text{Turnover rate}\left(p\right)=\frac{\text{nurse turnover}\left(\text{number of nurses}\right)}{\text{total number of nurses sampled }\left(N\right)}. \end{array}$$

The standard error (SE) of the nurse turnover rate [24] was computed by using the following formula:

(2) $$\begin{array}{c} \text{SE}=\sqrt{\frac{p\left(1-p\right)}{n}}. \end{array}$$

### 2.6. Data Analysis

Due to the heterogeneity between the studies, a random effect model with a confidence interval (CI) of 95% was used [25]. All of the statistical analyses were performed by using STATA 17.

Statistical heterogeneity was assessed by using the *I*^2^ statistic, with *I*^2^ values of 25%, 50%, and 75% indicating mild, moderate, and high heterogeneity, respectively. An *I*^2^ value lower than 50% was considered to be acceptable [26]. Subgroup analyses were conducted for region, sample size (<1,653 and ≥1,654 participants), time of survey initiation (2000–2008 and 2009–2019), year of publication (2003–2014 and 2015–2023), data sources, and departments. Moreover, to determine the factors associated with the nurse turnover rate, the hazard ratios (HRs) and 95% confidence intervals (CIs) of the influencing factors were combined and examined in a random effects model. Group analysis by sex, labour union, hospital size, work environment satisfaction, and job content satisfaction was performed to test the influence of relevant factors. Publication bias was assessed by using funnel plots and Begg's test [27], and the robustness of the findings was determined via sensitivity analysis. A *P* < 0.05 (two-sided test) was considered to indicate statistical significance.

## 3. Results

### 3.1. Summary of the Search Results

**Figure 1:**
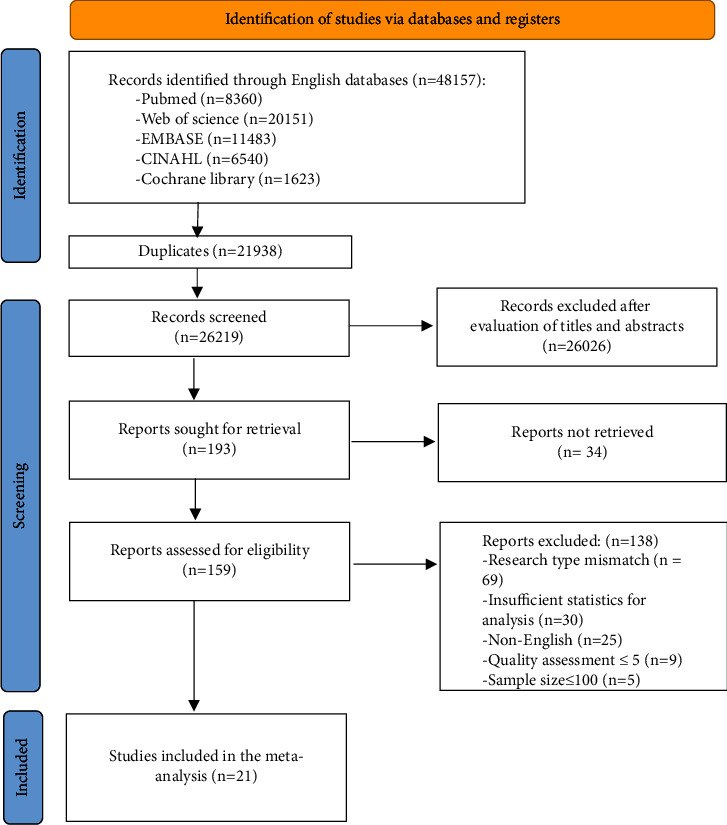
Flow diagram of study selection.

Twenty-one studies were ultimately included in this meta-analysis. Initially, 48,157 records were retrieved from 5 databases, of which 21,938 duplicates were removed, thus resulting in 26,219 studies. After screening the titles and/or abstracts, 193 studies were included in the full-text evaluation. According to the exclusion criteria, 69 studies were excluded due to inconsistent study type, 25 studies were excluded for being published in languages other than English, 30 studies were excluded for having incomplete relevant data, 5 studies were excluded for having a sample size <100, and 9 studies were excluded for being low-quality studies. Ultimately, the meta-analysis included 21 studies [16, 22, 28–46]. The details of the screening process are shown in Figure 1.

### 3.2. Description of the Included Studies

The characteristics of the 21 studies that were included in this meta-analysis are presented in Table 1. The studies were published between 2003 and 2023. These studies included 213,314 nurses from 14 countries with sample sizes ranging from 226 to 96,158. The age of the study participants ranged from 23 years to 46.9 years. Regarding the geographical regions, the number of studies conducted in Asia (8) and North America (7) was close. Conversely, Oceania, Europe, and Africa are represented equally, each contributing two studies to the analysis.

### 3.3. Methodological Quality

In this study, a methodological quality assessment of 21 studies was conducted by using the risk of bias assessment tool developed by Hoy and colleagues, and details of the assessment process are provided in Table 2. Five studies exhibited high quality, achieving scores ranging from 9 to 10. Sixteen studies were of moderate quality, with scores between 6 and 8 points. The average quality score of the 21 studies was 7.

### 3.4. Pooled Prevalence of Nurses' Turnover

The distribution of turnover rates across the studies ranged from 8% to 36.6%. A study conducted in Jordan had the highest turnover rate, and a study conducted in the United States had the lowest turnover rate. Random effects models were used due to the significant degree of heterogeneity (*I*^2^ = 98.61%, *P* < 0.01).

**Figure 2:**
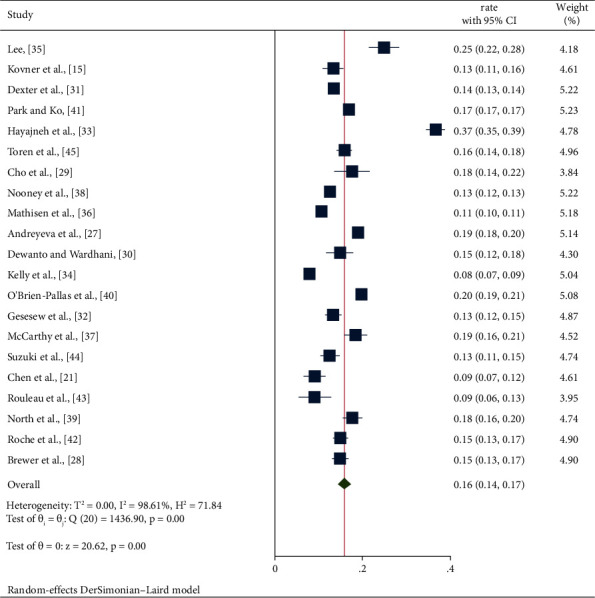
Pooled random effects turnover rate and 95% confidence intervals.

**Figure 3:**
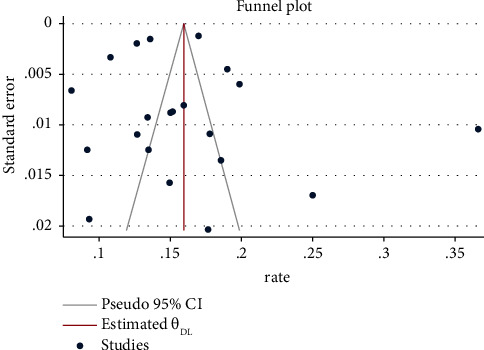
Funnel plot of the incidence of turnover.

The meta-analysis demonstrated a global nurse turnover rate of 16% (95% CI: 0.14, 0.17).  Figure 2 shows the forest plots obtained from the meta-analysis. The funnel plot showed asymmetry (Figure 3), and Begg's test showed that there was no significant publication bias (*P*=0.695).

### 3.5. Sensitivity Analyses

**Figure 4:**
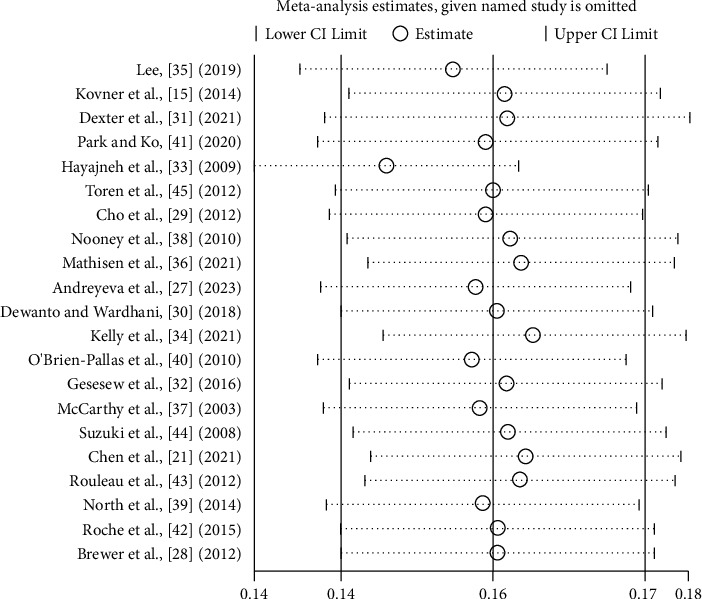
Forest plot of the sensitivity analysis.

A sensitivity analysis was conducted on the 21 articles included (Figure 4) using Stata 17 to assess the robustness of the meta-analysis results. After eliminating each single study, there was no significant difference between the combined effect value and the total combined value, thus indicating that the results of this study had good stability.

### 3.6. Subgroup Analyses

**Figure 5:**
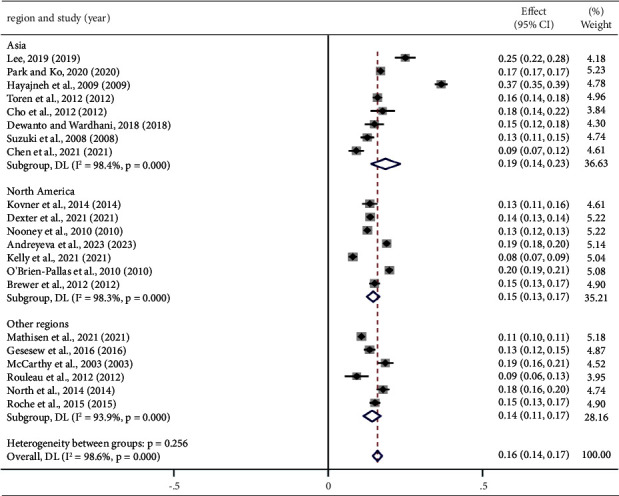
According to the region, the turnover rate of nurses.

To explore the sources of heterogeneity, we performed subgroup analyses based on region, sample size, study start time, publication year, data sources, and departments (Table 3). The study demonstrated that the turnover rate of nurses in Asia (19%; 95% CI: 0.14, 0.23) was greater than that in North America (15%; 95% CI: 0.13, 0.17), but there was no significant difference between the subgroups. Similarly, no significant differences were found across varying sample sizes (Figure 5).

**Figure 6:**
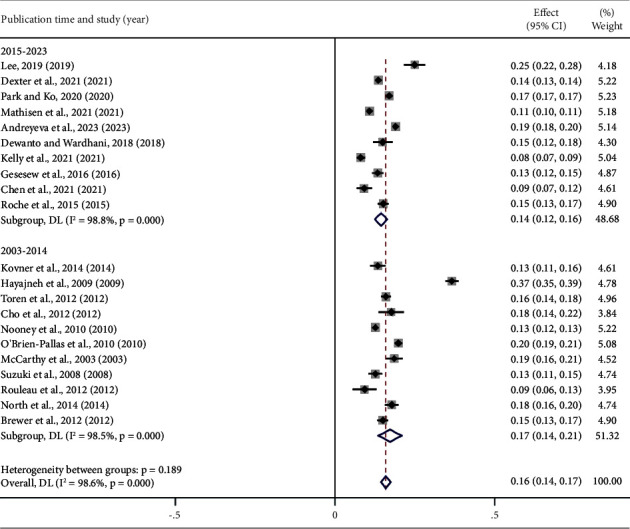
According to the publication time, the turnover rate of nurses.

According to a subgroup analysis by year of publication, the global nurse turnover rate was higher from 2003 to 2014 at 17% (95% CI: 0.14, 0.21) compared to 14% from 2015 to 2023 (95% CI: 0.12, 0.16). Analysis by study start time showed turnover rates of 18% (95% CI: 0.14, 0.21) from 2000 to 2008 and 13% (95% CI: 0.12, 0.16) from 2009 to 2019, with this difference being statistically significant (*P*=0.025) (Figure 6).

**Figure 7:**
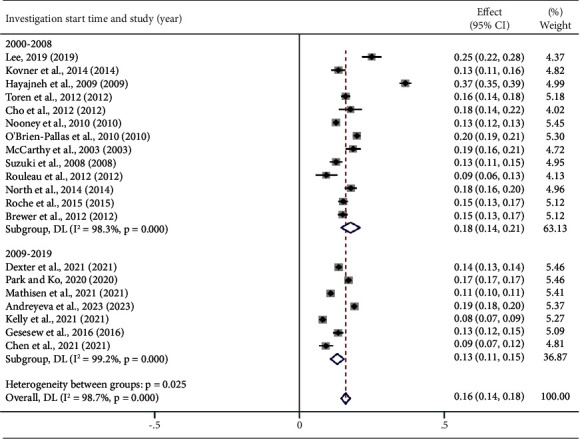
According to the investigation start time, the turnover rate of nurses.

Subgroup analysis by the data source revealed that global nurse turnover rates were higher in studies using databases at 18% (95% CI: 0.16, 0.20) compared to those using hospital data at 12% (95% CI: 0.10, 0.15), a statistically significant difference (*P* < 0.001) (Figure 7).

**Figure 8:**
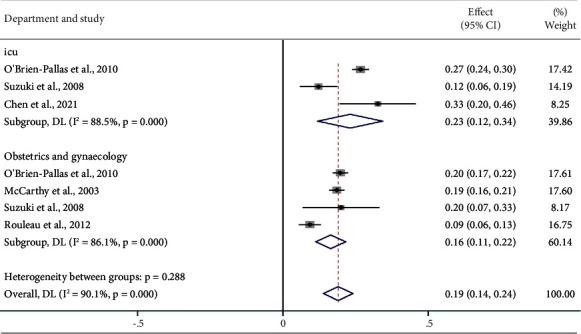
According to department, the turnover rate of nurses.

Five articles reported on the turnover rate of department nurses [22, 38, 41, 44, 45]. According to the subgroup analysis by department, the turnover rate of ICU nurses was 23% (95% CI: 12%–34%) and the turnover rate of obstetrics and gynecology nurses was 16% (95% CI: 11%–22%) (Figure 8).

### 3.7. Factors Associated with Nurse Turnover

Four articles reported on the factors associated with turnover [30, 36, 42, 45]. The analysis of group differences demonstrated that the main factors included sex, labour unions, hospital size, work environment satisfaction, and job content satisfaction (Table 4). Specifically, nurses who were not members of trade unions (HR = 0.62, 95% CI: 0.51–0.77), worked in smaller hospitals (HR = 2.99, 95% CI: 2.89–3.10), were dissatisfied with their work environment (HR = 2.12, 95% CI: 1.40–3.23), or were dissatisfied with their work content (HR = 1.76, 95% CI: 1.21–2.55) were more likely to leave.

## 4. Discussion

Nurse turnover is an important issue that has attracted widespread attention from health care institutions worldwide. The World Health Organization has called for increased investment in human resources for nurses and advocates for policy attention and support through education, training, regulation, and employment systems [47]. Although the reported worldwide nurse turnover rates are inconsistent, higher nurse turnover rates will undoubtedly have a serious negative impact on the health care system, not only causing greater economic burdens for medical institutions but also potentially having adverse effects on the allocation of nursing human resources, nurses' job satisfaction, and patients' health outcomes [48]. Consequently, this study represents a leading meta-analysis that provides a comprehensive estimate of the prevalence of nursing turnover from a global perspective.

### 4.1. Combined Prevalence of Nurses' Turnover

This study synthesizes results from 21 studies published between 2003 and 2023, involving 213,314 nurses from 14 countries. It found that nurses' turnover rates range from 8% to 36.6%, with a global combined rate of 16% (95% CI: 14%–17%). These data are similar to the 18% nurse turnover rate reported in 2024 [49]. According to other studies, the turnover rate of nurses is generally greater than that of other professionals in the health care field [50] thus indicating that the issue of staff turnover may be more pronounced in the nursing industry. The high overall prevalence rate once again underscores that nurses' turnover rate is a concern warranting attention, with an urgent need for additional efforts to mitigate attrition in this workforce. However, due to variations in the definitions and measurement methods of nurse turnover rates among the studies in this meta-analysis, future research requires a unified definition and standardized measurement approach to more accurately assess and compare nurse turnover rates. Moreover, given that differences in health care systems, employment settings, cultural contexts, and professional standards across countries could affect the outcomes, significant heterogeneity may exist in the pooled results. Therefore, the combined prevalence estimates should be interpreted with caution. Furthermore, identifying the factors that could influence the turnover rate among nurses is also a critical issue.

Thus, this study conducted subgroup analyses to investigate the sources of the observed high heterogeneity in differences.

### 4.2. Geographical Region

This meta-analysis showed that the turnover rate of nurses in Asia (19%) was higher than in North America (15%), which may be attributed to different economic and cultural systems, as well as the management and operation modes of hospitals. Economically developed areas often offer more employment options, and nurses may find jobs with higher pay, better working conditions, or more prospects. Moreover, the lower nurse turnover rate in North America may be due to its health care systems placing greater emphasis on this issue, as evidenced by more extensive research conducted there. Another major reason may be related to the shortage of nurses in Asia. With respect to the global population, Asia ranks first and its nurse-patient ratio is seriously unbalanced. A survey demonstrated that the density of nurses in Asia is lower than the global density [51], and a shortage of nurses directly increases the workload of working nurses and negatively affects their job satisfaction, thus increasing the possibility of nurse turnover [52, 53]. In contrast, the limited research on nurse turnover in Africa (54.4%) suggests less attention to the issue in these regions. More studies in the future could help refine these estimates globally.

### 4.3. Time of Publication and Study Start Time

The original studies that were included in this meta-analysis were published between 2003 and 2023. Subgroup analysis demonstrated that the global combined nurse turnover rate from 2015 to 2023 was lower than that from 2003 to 2014. Even for subgroup analysis based on study start time, the global combined nurse turnover rate between 2009 and 2019 was significantly lower than that between 2000 and 2008. The abovementioned studies show that the turnover rate of nurses in the 14 countries that were included in this study has exhibited a declining trend in recent years. This trend aligns with the World Health Organization's repeated emphasis on the importance of nurses, advocating the need to unleash their true potential and ensure that they have the resources and support to meet global health needs [3].

Considering that numerous studies have shown that nurse turnover significantly impacts hospital budgets and health care expenditure costs [43, 48], a growing number of countries worldwide are seeking to maintain the long-term stability of the nurse workforce by reducing nurse turnover. On the other hand, the education and professional skill levels of nurses have notably increased over the past two decades, leading to an enhanced sense of professional identity, which has been identified as one of the main factors in the reduction of turnover rates [54, 55]. Simultaneously, governments and health systems have become increasingly aware of the vital role of nurses in improving patient health outcomes; thus, they have introduced policies and systems to actively improve the practice environment of nurses and improve their treatment and satisfaction [53, 56].

### 4.4. Work Department

Subgroup analysis demonstrated that the turnover rate was 23% for nurses working in ICUs and 16% for nurses working in obstetrics and gynecology departments. Nurses working in ICUs and obstetrics and departments have heavy workloads and long working hours; moreover, they frequently work overtime, are in a state of stress for a long period of time, and bear a considerable psychological burden. Studies have shown that nurses working in ICUs have high levels of stress, dissatisfaction, and job burnout [57, 58], and these factors are the main causes of nurse turnover [59]. These results suggest that nursing managers should prioritize the job satisfaction and physical and mental health of nurses in ICUs and obstetrics and gynecology departments. Appropriate actions, such as reasonable scheduling and increasing staffing, should be taken to reduce turnover rates in these areas.

### 4.5. Factors Associated with Turnover

Some studies have demonstrated that nurses who were not union members, who worked in smaller hospitals, and who were dissatisfied with the work environment and content were more inclined to depart.

Firstly, hospitals lacking union support may fall short in protecting employee rights, benefits, the work environment, and career development, all of which could lead to higher nurse turnover rates. Additionally, smaller hospitals often experience limitations in offering career development opportunities, creating a positive work environment, and allocating resources. These limitations may include limited advancement paths, fewer professional training resources, lower social recognition, and greater job stress. These factors collectively may lead nurses to feel constrained in their professional growth, thereby motivating them to seek healthcare institutions that offer broader career prospects and superior working conditions. Research by Park and Ko supports this viewpoint, thus indicating that nurses particularly value opportunities for professional development and the quality of the work environment when evaluating potential job opportunities [42]. Therefore, to attract and retain nursing talent, small hospitals need to improve these critical factors; for instance, they can establish clear career development plans, offer regular professional training, improve working conditions, increase resource allocation, and enhance social recognition of nursing work. Through these measures, small hospitals can create an environment more conducive to nurses' professional growth and personal development.

Moreover, nurse turnover and lower job satisfaction are closely related, which supports the findings of Li et al. [60]; specifically, nurses want to have a cohesive, supportive, and independent practice environment. Hence, it is imperative for hospital managers to establish a practice atmosphere that fosters a cohesive team and supportive management. Finally, it would be more appropriate to be conservative in interpreting turnover-related factors because these meta-analyses are based on a limited number of studies. However, these findings provide important insights for future studies. These factors may greatly affect nurses' resignation, thus motivating researchers to conduct relevant large sample experiments in the future.

Obviously, nurse turnover is also influenced by other factors. Jones et al. categorized the reasons for leaving into internal and external factors. Internal reasons primarily include seeking opportunities for career development and advancement, job burnout, a tense work environment, and a lack of good leadership and management. External reasons mainly include nurses pursuing better compensation and benefits, experiencing high job stress, and having inadequate staffing [61]. Lee categorized the main reasons for nurse turnover into personal, hospital, and profession-specific factors. Among personal factors, the turnover rate among men is greater than that among women, which is a result that may partly be attributed to gender imbalance because the nursing profession is still predominantly occupied by women. Additionally, the education level also plays a significant role in personal factors, as nurses with higher education may have greater capabilities to identify and access information about employment opportunities that are available in the current job market, thus resulting in better career planning. In addition to organizational factors within hospitals, career prospects, autonomy, social esteem, and interpersonal relationships (the satisfaction derived from the profession itself) are also factors affecting nurse turnover [35, 36].

Hayes et al. conducted a detailed analysis of the factors influencing nurse turnover and categorized them into three main areas: organizational factors, personal factors, and factors related to career development and economics. Among the organizational factors, four key points were emphasized: high workload intensity, ongoing stress and burnout, inadequate leadership abilities of managers, lack of empowerment, and high levels of role ambiguity and role conflict are all associated with higher nurse turnover rates. In terms of personal factors, an inverse relationship was reported between age, nurses with family responsibilities (e.g., dependent children or relatives), years of nursing experience, length of time in the position, and likelihood of leaving. Finally, in terms of career development and economic factors, nurses may choose to leave their current positions if they perceive better career development opportunities and compensation benefits that are available elsewhere [62]. To reduce the turnover rate of nurses, health care institutions need to comprehensively consider these factors and take appropriate measures to address them.

### 4.6. Strengths and Limitations

Overall, the study conducted a comprehensive search of the relevant global literature to minimize the risk of omitting studies due to selection bias. Furthermore, through the use of rigorous methodological procedures and statistical analysis, data were collected and aggregated from 213,314 nurses from 14 countries, thus providing a scientific basis for policy makers worldwide who want to address employment stability in the nursing industry. Furthermore, both sensitivity and subgroup analyses were conducted to examine the potential sources of heterogeneity to enhance the rigor and reliability of the study.

In interpreting and applying the findings of this study, certain limitations must be considered. First, to utilize more accurate turnover rate data, certain studies were excluded because they only reported turnover rate, without providing the total employee number or the specific count of individuals who had left. This exclusion may have slightly influenced the study outcomes. Second, there was no clear, consistent definition of “turnover” in the included studies, which potentially led to the possibility of slight bias in the study findings. Moreover, due to the limited information presented by the original studies, there were insufficient data to explore the reasons and factors influencing turnover in this study. Finally, since the majority of the included studies were written in English, interpretations of the research findings should be approached with caution.

Therefore, future studies should include more detailed information on occupation-related, demographic, and family-social aspects, especially regarding the turnover rate of nurses working in different departments with different academic qualifications, ages, marital statuses, and durations of employment, which will be helpful for in-depth analysis of related factors. In addition, when experts and scholars evaluate nurse turnover, there should be a clearer definition of voluntary resignation and dismissal by medical institutions, which will facilitate a more comprehensive comparison. Given that there are few research articles on the nurse turnover rate in various countries that mainly focus on nurses' turnover intentions, this study suggests that research on groups of nurses who actually leave their positions in the future should be increased. Only by truly understanding the factors influencing turnover in nurses who have left their positions can more accurate and more specific intervention measures or more reasonable policies and systems be formulated to reduce the turnover of nurses.

## 5. Conclusion

This study analysed existing published studies related to nurse turnover worldwide and demonstrated that the global nurse turnover rate was approximately 16%, thus implying an urgent need for efforts to reduce nurse turnover. High nurse turnover rates were observed in Asia (19%), ICUs (23%), and obstetrics and gynecology departments (16%). This emphasizes the need for health policy makers and nursing managers, particularly in Asia, to focus on reducing turnover in ICU and obstetrics and gynecology settings. The findings of this study underscore the urgent need for future intervention research aimed at reducing turnover among nursing staff, thereby enhancing the quality of patient care. However, research on the turnover rates of this population is relatively new and limited in some continents; therefore, further studies are necessary to more accurately measure the prevalence among this group.

## 6. Implication for Nursing Management

The study result shows that approximately 16% of nurses have experienced turnover, indicating an urgent need for efforts to reduce turnover. It provides a more comprehensive and objective understanding of nurse turnover for health care policy makers, hospital managers, and nursing leaders. It is suggested that all medical and health institutions actively adopt relevant systems and supporting policies that can reduce the turnover of nurses and promote a more harmonious, healthy, and safe occupational environment for nurses to strengthen the stability and sustainable development capacity of the nurse workforce. To reduce the turnover rate among nurses, it is essential to consider a comprehensive approach that addresses various factors such as the work environment, compensation and benefits, career development, workload, and psychological stress. For instance, human resources should be allocated rationally to reduce nurses' work stress and increase job satisfaction, adopting scheduling systems and work principles that promote nurses' physical and mental health. Additionally, establishing an attractive hospital culture can enhance nurses' sense of belonging and provide personalized, diverse career development opportunities, thereby improving their satisfaction with the professional environment. Furthermore, strengthening public education to increase awareness and understanding of nursing roles will enhance nurses' professional identity and motivate them actively in their work.

## Figures and Tables

**Table 1:** Characteristics of the included studies.

No	Author (year)	Country	Year	Study design	Sampling method	Survey method	Sample size	Turnover rate (%)	Data source	Mean age (years)	Female (%)
1	Andreyeva et al. [28] (2023)	US	2016-2017	CS	General survey	NR	7,634	19	Database	39.5	91.3
2	Brewer et al. [29] (2012)	US	2006-2007	LS	Random	E-mail	1,653	15	Hospital	32	91
3	Chen et al. [22] (2021)	China	2017-2018	Co	Convenience sampling	Questionnaire	553	8.9	Hospital	30	91.1
4	Cho et al. [30] (2012)	Korea	2006–2008	CS	Multistage stratified sampling	Questionnaire	351	17.7	Database	24.2	96
5	Dewanto and Wardhani [31] (2018)	Indonesia	NR	CS	NR	NR	515	15	Hospital	NR	67.6
6	Dexter et al. [32] (2021)	US	2016-2017	CS	Random	Questionnaire	50,273	13.6	Database	NR	NR
7	Gesesew et al. [33] (2016)	Ethiopia	2009–2014	CS	Simple random sampling	Questionnaire	1,358	13.4	Hospital	NR	NR
8	Hayajneh et al. [34] (2009)	Jordanian	2006-2007	CS	Random	Telephone survey	2,126	36.6	Database	NR	48.5
9	Kelly et al. [35] (2021)	US	2018-2019	CS	General survey	E-mail	1,688	8	Hospital	39.9	89
10	Kovner et al. [16] (2014)	US	2004-2005	LS	Random	E-mail	750	13.4	Database	NR	NR
11	Lee [36] (2019)	Korea	2008–2010	LS	Stratified random sampling	NR	652	25	Database	24.8	90.5
12	Mathisen et al. [37] (2021)	Danish	2014	Co	General survey	NR	8,768	10.8	Database	NR	NR
13	McCarthy et al. [38] (2003)	Ireland	2000-2001	CS	General survey	Questionnaire	834	18.5	Database	NR	NR
14	Nooney et al. [39] (2010)	US	2004	CS	General survey	Telephone survey	26,472	12.6	Database	46.9	94.1
15	North et al. [40] (2014)	New Zealand	2005–2010	Co	NR	NR	1,236	17.8	Database	30.3	94
16	O'Brien-Pallas et al. [41] (2010)	Canadian	2005-2006	CS	General survey	NR	4,481	19.9	Database	38.9	NR
17	Park and Ko [42] (2020)	Korea	2011–2016	Co	General survey	E-mail	96,158	17	Database	NR	97.6
18	Roche et al. [43] (2015)	Australia	2008–2010	LS	NR	NR	1,673	15.1	Hospital	39.2	NR
19	Rouleau et al. [44] (2012)	Senegal	2007-2008	LS	NR	Questionnaire	226	18	Hospital	40.4	NR
20	Suzuki et al. [45] (2008)	Japan	2003–2005	Co	NR	Questionnaire	923	12.7	Hospital	23	96.4
21	Toren et al. [46] (2012)	Israel	2008-2009	CS	Random	Telephone survey	2,098	16	Database	43	88

LS: longitudinal study; CS: cross-sectional study; Co: cohort study; Year: year of data collection; NR: not reported.

**Table 2:** Quality Assessment for included studies.

Author (year)	Risk of bias assessment tool item										
	1	2	3	4	5	6	7	8	9	10	Total score
Andreyeva et al. [28] (2023)	1	1	1	0	1	1	0	1	1	1	8
Brewer et al. [29] (2012)	1	1	1	0	1	1	1	1	1	1	9
Chen et al. [22] (2021)	1	1	1	1	1	1	0	1	1	1	9
Cho et al. [30] (2012)	1	1	1	0	1	1	0	1	0	0	6
Dewanto and Wardhani [31] (2018)	1	0	0	0	1	0	1	1	1	1	6
Dexter et al. [32] (2021)	1	1	1	0	1	0	0	1	1	0	6
Gesesew et al. [33] (2016)	1	1	1	1	1	0	0	1	1	1	8
Hayajneh et al. [34] (2009)	1	1	1	1	1	1	1	1	1	1	10
Kelly et al. [35] (2021)	1	0	0	0	1	0	1	1	1	1	6
Kovner et al. [16] (2014)	1	1	1	0	1	1	1	0	1	1	8
Lee [36] (2019)	1	1	1	0	1	0	0	1	1	0	6
Mathisen et al. [37] (2021)	1	1	1	1	1	1	0	1	1	1	9
McCarthy et al. [38] (2003)	1	1	1	1	1	0	0	1	1	1	8
Nooney et al. [39] (2010)	1	1	1	0	1	1	0	1	0	0	6
North et al. [40] (2014)	1	0	0	1	1	0	0	1	1	1	6
O'Brien-Pallas et al. [41] (2010)	1	1	1	0	1	0	0	1	1	0	6
Park and Ko [42] (2020)	1	1	1	1	1	1	0	1	1	1	9
Roche et al. [43] (2015)	1	0	0	0	1	1	1	1	1	0	6
Rouleau et al. [44] (2012)	1	0	0	0	1	1	0	1	1	1	6
Suzuki et al. [45] (2008)	1	0	0	1	1	0	0	1	1	1	6
Toren et al. [46] (2012)	1	1	1	0	1	1	0	1	1	1	8

**Table 3:** Subgroup analysis of the overall turnover rate.

Subgroup	*k*	Turnover rate (%)	95% CI		*I* ^2^ (%)	*P* value	*P* value across subgroups
			Lower	Upper			
Area						
Asia	8	19	0.14	0.23	98.4	<0.001	0.256
North America	7	15	0.13	0.17	98.3	<0.001	
Other regions	6	14	0.11	0.17	93.9	<0.001	
Sample size						
<1,653	10	15	0.12	0.17	88.9	<0.001	0.183
≥1,654	11	17	0.15	0.19	99.3	<0.001	
Investigation start time						
2000–2008	13	18	0.14	0.21	98.3	<0.001	0.025
2009–2019	7	13	0.12	0.16	99.2	<0.001	
Publication time						
2003–2014	11	17	0.14	0.21	98.5	<0.001	0.189
2015–2023	10	14	0.12	0.16	98.8	<0.001	
Data source						
Databases	13	18	0.16	0.20	99.1	<0.001	0.000
Hospitals	8	12	0.10	0.15	90.4	<0.001	
Department						
ICU	3	23	0.12	0.34	88.5	<0.001	0.288
Obstetrics and gynecology	4	16	0.11	0.22	86.1	<0.001	

**Table 4:** Meta-analysis of risk factors associated with nurse turnover.

Associated factors	Studies (*n*)	HR	Lower limit	Upper limit	*I* ^2^ (%)	*P* for heterogeneity
Female (ref: male)	2	0.91	0.51	1.62	90.5	0.001
Labour union (no)	2	0.62	0.51	0.77	0.00	0.561
Hospital size (vs. large)						
Small	2	2.99	2.89	3.10	0.00	0.743
Medium	2	1.63	1.59	1.68	0.00	0.941
Workplace dissatisfaction (vs. satisfied or neutral)	3	2.12	1.40	3.23	68.9	0.040
Work content dissatisfaction (vs. satisfied or neutral)	2	1.76	1.21	2.55	50.6	0.155

## Data Availability

The datasets supporting this meta-analysis are from previously reported studies and datasets, which have been cited. The processed data are available from the corresponding author upon request.

## Additional Points

*Implications for Nursing Management/Contribution of the Paper*. The shortage of nurses and high turnover rates pose significant challenges for healthcare organizations worldwide. Future research on nurse attrition should incorporate more comprehensive occupation-related, demographic, and family-social information, particularly by examining attrition patterns among nurses in different departments, educational backgrounds, age groups, marital statuses, and years of experience. It is crucial to clearly differentiate between voluntary attrition and resignation to provide a solid foundation for healthcare policy makers, administrators, and care leaders in formulating relevant policies. This study has several implications for nursing management. For example, managers must prioritize nurse retention efforts by strategically planning human resources to ensure an adequate nursing workforce that can meet patient needs; moreover, they should offer career advancement opportunities and foster professional development while cultivating a strong team spirit and fostering a positive organizational culture to enhance nurses' sense of belongingness and loyalty. By stabilizing the nursing team through these measures, the quality of nursing services can be improved, thus ultimately leading to better patient care.

## Supplementary Materials

Supplementary Materials Appendix A: we searched PubMed, Web of Science, Embase, CINAHL, and Cochrane Library databases. The search strategy used for each database is provided in Appendix A.
