# Correction to “Global Prevalence of Nurse Turnover Rates: A Meta-Analysis of 21 Studies from 14 Countries”

**DOI:** 10.1155/jonm/9754202

**Published:** 2025-11-17

H. Ren, P. Li, Y. Xue, W. Xin, X. Yin, and H. Li, “Global Prevalence of Nurse Turnover Rates: A Meta-Analysis of 21 Studies from 14 Countries,” *Journal of Nursing Management*, 2024, https://doi.org/10.1155/2024/5063998.

In the article, there is an error in the abstract:

“This meta-analysis analysed the literature published from January 2020 to February 2023 and demonstrated that the global nurse turnover rate was 16%.”

Should read

“This meta-analysis analysed the literature published from January 2000 to February 2023 and demonstrated that the global nurse turnover rate was 16%.”

We apologize for this error.

