# Supplementary material for: Global Prevalence of Nurse Turnover Rates: A Meta-Analysis of 21 Studies from 14 Countries
Source: J Nurs Manag. 2024 Jun 12;2024:5063998. doi: 10.1155/2024/5063998 (PMC11919231; doi:10.1155/2024/5063998)
Supplement: Supplementary Materials — Appendix A: we searched PubMed, Web of Science, Embase, CINAHL, and Cochrane Library databases. The search strategy used for each database is provided in Appendix A. [file 5063998.f1.docx]

**Appendix a:** **Developed search strategy**

| **N** | **Datebase** | **Index and Keyword Terms** | **Results** |
| --- | --- | --- | --- |
| **1** | Pubmed | (nurs*[Title/Abstract]) AND (attrition[Title/Abstract] OR leav*[Title/Abstract] OR turnover[Title/Abstract] OR quit[Title/Abstract]) | 8360 |
| **2** | Web of Science | topic=（（attrition OR leav* OR turnover OR quit）AND nurs*） | 20151 |
| **3** | Embase | \| 1 \| 'nurse'/exp \| \| --- \| --- \| \| 2 \| nurs*:ti,ab,kw \| \| 3 \| #1 OR #2 \| \| 4 \| attrition:ti,ab,kw OR leav*:ti,ab,kw OR turnover:ti,ab,kw OR quit:ti,ab,kw \| \| 5 \| #3 AND #4 \| | 11483 |
| **4** | CINAHL | \| 1 \| (MH "Nurses") OR ( TI nurs* ) OR ( AB nurs* ) \| \| --- \| --- \| \| 2 \| ( TI ( attrition OR leav* OR turnover OR quit ) OR AB ( attrition OR leav* OR turnover OR quit ) ) OR (MH "Personnel Turnover") \| \| 3 \| 1 AND 2 \| | 6540 |
| **5** | Cochrane | \| 1 \| MeSH descriptor: [Nurses] explode all trees \| \| --- \| --- \| \| 2 \| (nurs*):ti,ab,kw \| \| 3 \| MeSH descriptor: [Personnel Turnover] explode all trees \| \| 4 \| (attrition OR leav* OR turnover OR quit):ti,ab,kw \| \| 5 \| #1 OR #2 \| \| 6 \| #3 OR #4 \| \| 7 \| #5 AND #6 \| | 1623 |

Abbreviations: AB=Abstract; ab,ti: Title or Abstract; ti,ab,kw: Title, Abstract or Author keywords;
